# Deep learning for automated segmentation and counting of hypocotyl and cotyledon regions in mature *Pinus radiata* D. Don. somatic embryo images

**DOI:** 10.3389/fpls.2024.1322920

**Published:** 2024-03-01

**Authors:** Sam J. Davidson, Taryn Saggese, Jana Krajňáková

**Affiliations:** ^1^ Data and Geospatial Intelligence, New Zealand Forest Research Institute (Scion), Christchurch, New Zealand; ^2^ Forest Genetics and Biotechnology, New Zealand Forest Research Institute (Scion), Rotorua, New Zealand

**Keywords:** deep learning, semantic segmentation, instance segmentation, somatic embryo, embryo morphology, plant phenotyping, automated counting

## Abstract

In commercial forestry and large-scale plant propagation, the utilization of artificial intelligence techniques for automated somatic embryo analysis has emerged as a highly valuable tool. Notably, image segmentation plays a key role in the automated assessment of mature somatic embryos. However, to date, the application of Convolutional Neural Networks (CNNs) for segmentation of mature somatic embryos remains unexplored. In this study, we present a novel application of CNNs for delineating mature somatic conifer embryos from background and residual proliferating embryogenic tissue and differentiating various morphological regions within the embryos. A semantic segmentation CNN was trained to assign pixels to cotyledon, hypocotyl, and background regions, while an instance segmentation network was trained to detect individual cotyledons for automated counting. The main dataset comprised 275 high-resolution microscopic images of mature *Pinus radiata* somatic embryos, with 42 images reserved for testing and validation sets. The evaluation of different segmentation methods revealed that semantic segmentation achieved the highest performance averaged across classes, achieving F1 scores of 0.929 and 0.932, with IoU scores of 0.867 and 0.872 for the cotyledon and hypocotyl regions respectively. The instance segmentation approach demonstrated proficiency in accurate detection and counting of the number of cotyledons, as indicated by a mean squared error (MSE) of 0.79 and mean absolute error (MAE) of 0.60. The findings highlight the efficacy of neural network-based methods in accurately segmenting somatic embryos and delineating individual morphological parts, providing additional information compared to previous segmentation techniques. This opens avenues for further analysis, including quantification of morphological characteristics in each region, enabling the identification of features of desirable embryos in large-scale production systems. These advancements contribute to the improvement of automated somatic embryogenesis systems, facilitating efficient and reliable plant propagation for commercial forestry applications.

## 1 Introduction

The propagation of conifers, such as Pinus *radiata* D. Don., holds significant importance for meeting global timber demands, reforestation efforts, and the preservation of natural ecosystems. With the growing need to ensure sustainable and efficient conifer propagation, advanced techniques for mass propagation of high-quality genetics are of high importance.

Somatic embryogenesis (SE) is an advanced developmental method by which plants can regenerate bipolar structures from a somatic cell (Méndez-Hernández et al., 2019). These bipolar structures in their development and morphological features resemble their zygotic counterparts (von Arnold et al., 2020). In conifers, SE is the preferable method of propagation due to the possibility of long-term cryo-storage of the embryogenic tissue. It is a multi-step process, starting with induction of embryogenic tissue, followed by proliferation and formation of early somatic embryos in the presence of auxins and cytokinins. The development continues with the change of nutrient media. Addition of abscisic acid enhances the maturation of somatic embryos which is followed by germination and regeneration of the intact plantlet (Stasolla and Yeung, 2003). The success of plantlet regeneration is dependent on the proper execution of each step and several chemical and physical stimuli may be employed (Filonova et al., 2002; von Arnold et al., 2020). In the end of the maturation process a population of somatic embryos is obtained consisting of a mixture of pre-cotyledonary and cotyledonary somatic embryos and remains of the proliferating tissue as the maturation process is unsynchronized (Stasolla and Yeung, 2003).

The selection of high quality mature somatic embryos and the transfer into germination conditions is traditionally done manually by trained personnel. A trained tissue culturist will select embryos from the population based on qualitative assessment of morphological features such as size, shape, and the number of cotyledons. Since this is a very labour-intensive process, the latest advances in the tissue culture technologies focus on the automation of this process, either by using fluidics systems or picking robots for the isolation of individual mature embryos from the surrounding tissue (Find and Krogstrup, 2008; Egertsdotter et al., 2019). Selection of high-quality somatic embryos requires fast decision making based on morphological criteria, with the somatic embryo either being accepted or rejected. The basis of this process is the accurate quantification of mature somatic embryo characteristics from imagery, providing the foundation for subsequent analysis. Several studies have used morphological features related to shape and size derived from images to assess germination potential or somatic embryo quality (Uozumi et al., 1993; Zhang et al., 1999; Le et al., 2021). The crucial first step in quantifying such characteristics is the automated delineation of the embryo boundary, including any specific regions of interest. The extracted data can provide valuable insights into the development of the embryos, as well as help improve the efficiency and success rates of the SE process.

For the past three decades, artificial intelligence techniques have been increasingly used in the tissue culture space; previously explored AI models have included artificial neural networks, neurofuzzy logic, support vector machines, decision trees and random forest (Hesami and Jones, 2020). Their applications include prediction of length, number of microshoots and roots, biomass prediction, optimization of environmental conditions, as well as automated somatic embryo and micro-shoot classification (Prasad and Gupta, 2008; Osama et al., 2015; Hesami and Jones, 2020).

Previous studies perform segmentation of somatic embryos using binary image thresholding to automatically segment mature somatic embryos in greyscale images and as a result can obtain the embryo area and boundary (Hamalainen and Jokinen, 1993; Hamalainen et al., 1993; Uozumi et al., 1993; Chi et al., 1996; Find and Krogstrup, 2008; Le et al., 2021). Binary image thresholding involves converting a greyscale or colour image into a black and white (binary) image, then selecting a threshold value and assigning pixel values based on whether they are above or below the chosen threshold. Le et al. (2021) use binary image thresholding in greyscale images of Norway spruce in their use of the fluidics system (Le et al., 2021). Similarly, Hamalainen et al. (1993) used a binary threshold in high contrast bottom view greyscale images of birch (*Betula pendula* Roth) somatic embryos (Hamalainen et al., 1993). Likewise (Find and Krogstrup, 2008), used a binary threshold on images of nordmanns fir (*Abies nordmanniana*) and sitka spruce (*Picea sitchensis*) somatic embryos. Uozumi et al., 1993 used the same for segmenting celery embryos (Uozumi et al., 1993). Chi et al. (1996) used a binary threshold to distinguish carrot somatic embryos from the background, followed by a thinning algorithm to remove open contours and noise generated in the acquisition process. These approaches achieved satisfactory segmentations as the images had very high levels of contrast between the objects and background. However, thresholding techniques are unable to distinguish between different regions containing similar spectral values such as the upper and lower part of the embryo or between cotyledons with almost identical spectral intensities. Additional challenges for pine somatic embryos included the size of the embryos, the wide variations in the number of cotyledons per embryo and the overlapping nature of the cotyledons, making them difficult to distinguish in a single lateral view image. These factors make our tasks challenging for traditional image processing methods and make our data a good candidate for more sophisticated methods which utilise deep learning.

Advancements in deep learning and computer vision have opened new possibilities for automating the analysis of somatic embryo images. Deep learning is a form of machine learning where a model is typically trained on a set of examples related to the specific task (Janiesch et al., 2021). In the task of image segmentation, training examples are provided in the form of images and their corresponding annotations, also known as masks in the computer vison community. Deep learning methodologies utilising convolutional neural networks (CNNs) are revolutionising the way various image-related tasks are solved. These networks are capable of automatically extracting complex spatial and structural information from images, enabling them to accurately differentiate between different objects, regions, or categories within an image (Yamashita et al., 2018). These capabilities provide several advantages over traditionally used somatic embryo image segmentation techniques such as pixel thresholding with size filtering. Thresholding methods strongly rely on pixel intensities being different between classes and ignores the spatial component of neighbouring pixels. Therefore, as we are interested in delineating regions belonging to the same pixel intensities, we have opted to not use thresholding-based methods which rely purely on differences in intensities. The complexity of CNNs allow them to consider clusters of pixels to learn relevant shapes, patterns, and structures at different levels of abstraction, similar to the way humans use vision. This enables them to distinguish different objects or categories in regions of similar spectral intensity. It also allows them to be far more robust to regions containing noise such as reflections, shadows, and out of focus areas. As a result, CNNs have achieved state-of-the-art performance in image classification, object detection, and segmentation tasks (Taye, 2023).

In biological microscopy, deep learning has demonstrated promising performance in a range of segmentation applications including semantic segmentation of human oocyte (Targosz et al., 2021), semantic and instance segmentation for cell nuclei (Caicedo et al., 2019) and semantic segmentation potato tuber (Biswas and Barma, 2020). Examples of plant phenotyping applications include semantic and instance segmentation for plant leaf detection and counting (Aich and Stavness, 2017; Giuffrida et al., 2018; Itzhaky et al., 2018; Jiang et al., 2019; Fan et al., 2022), semantic and instance segmentation for crop phenotyping (Jiang and Li, 2020), grapevine leaf semantic segmentation (Tamvakis et al., 2022), barley seed detection from instance segmentation (Toda et al., 2020) and many other applications (Kolhar and Jagtap, 2023).

In this work, we focus on two widely used variations of CNN based segmentation networks: semantic segmentation and instance segmentation. Semantic segmentation is a computer vision technique used to assign a class label to each pixel in an image, while instance segmentation distinguishes individual instances of the same class by using a box detection step followed by pixel-level segmentation. Both techniques enable automated delineation of class boundaries as well as the area of the image they occupy, therefore, we evaluate and compare them as potential solutions to this pixel-level segmentation task. However, as instance segmentation allows for the delineation of individual instances, we additionally evaluate its’ ability to predict the number of cotyledons. In deep learning, the categories of interest within the image are referred to as classes, which, for our images, are the hypocotyl and cotyledon regions. Long et al. (2015) proposed the Fully Convolutional Network (FCN) a variant of CNN, which significantly increased segmentation accuracy over previous segmentation approaches (Long et al., 2015). In the following years, FCNs paved the way for deep-learning-based semantic segmentation. Residual Network (ResNet) is the variant of FCN we employ in this work for both semantic and instance segmentation, for its proven ability to learn fine-grained segmentation tasks (He et al., 2016).

Several techniques exist for counting overlapping objects including deep learning approaches such as CNN instance segmentation networks (Toda et al., 2020) and CNN based regression networks (Giuffrida et al., 2018; Itzhaky et al., 2018), and non-deep learning approaches such as distance transform combined with the watershed algorithm (Itakura and Hosoi, 2018). We chose instance segmentation for its’ ease of implementation and proven ability to perform multi-class detection and segmentation, allowing for counting of objects and enabling us to obtain semantic segmentations of cotyledon and hypocotyl regions. Instance segmentation provides an additional level of information to semantic segmentation and can distinguish individual instances of regions belonging to the same class. Instance segmentation does this by using an initial box detection step, which assigns an ID to each individual instance before proceeding to segment pixels inside that box to obtain the instance boundary. This allows for a more detailed further analysis enabling for counting and, if desired, individual measurements per instance.

The number of cotyledons in coniferous species is a distinctive feature for discrimination and serves as a valuable parameter for assessing the efficacy of maturation protocols in somatic embryogenesis, a biotechnological method applied for the propagation of these species (Chandler, 2008; Wang and Ran, 2014). The variation in cotyledon number within a given gymnosperm species correlates with embryo size, which alters from year to year (Butts and Buchholz, 1940). In somatic embryos, there is a greater degree of variation in cotyledon number compared with zygotic embryos (Harrison and Von Aderkas, 2004), and this number is an indicator of maturity (Zhang et al., 1999). For instance, normally developed Douglas fir somatic embryos typically have 4 to 7 cotyledons, with numbers outside this range considered abnormal (Zhang et al., 1999). Somatic embryo development is regulated by timed applications of exogenous plant growth regulating substances (PGRs), and the germination potential is notably influenced, as only embryos possessing a sufficient number of cotyledons demonstrate successful germination.

There has been limited research on delineating the cotyledon region or individual cotyledons from the rest of the embryo in mature somatic embryo image analysis. Timmis et al. (2015) considered both cotyledon count and length from segmented images of Douglas-fir SE. Barry-Etienne et al. (2002) digitized coffee somatic embryo cotyledons in a scanner, then manually obtained cotyledonary area and filtered them into small, medium, and large categories based on area, before correlating sizes with conversion into plantlets. They found embryos with large cotyledons to have a significantly lower conversion rate compared to the smaller categories (Barry-Etienne et al., 2002). Delineating the cotyledon region allows for measuring morphological features such as cotyledon region length, width and area, as well as computing the ratio of cotyledon to hypocotyl area. These findings underscore the crucial role that the number of cotyledons plays in shaping the outcomes of somatic embryogenesis and highlight the need for further research in this area.

In this study we use convolutional neural networks for the automated analysis of mature somatic embryo for two key image analysis tasks. The first aim was to compare and investigate the potential of semantic segmentation and instance segmentation for automated segmentation of the hypocotyl and cotyledon regions in *Pinus radiata* mature somatic embryo images. Our second aim was to accurately predict the number of cotyledons per embryo from instance segmentation by obtaining individual cotyledon detections from instance segmentation. To the best of our knowledge, this is the first work using deep learning for the automated annotation of somatic embryo images of conifers and the first to use instance segmentation to detect individual cotyledons as a way of obtaining cotyledon counts.

## 2 Materials and methods

### 2.1 Plant material

**Figure 1:**
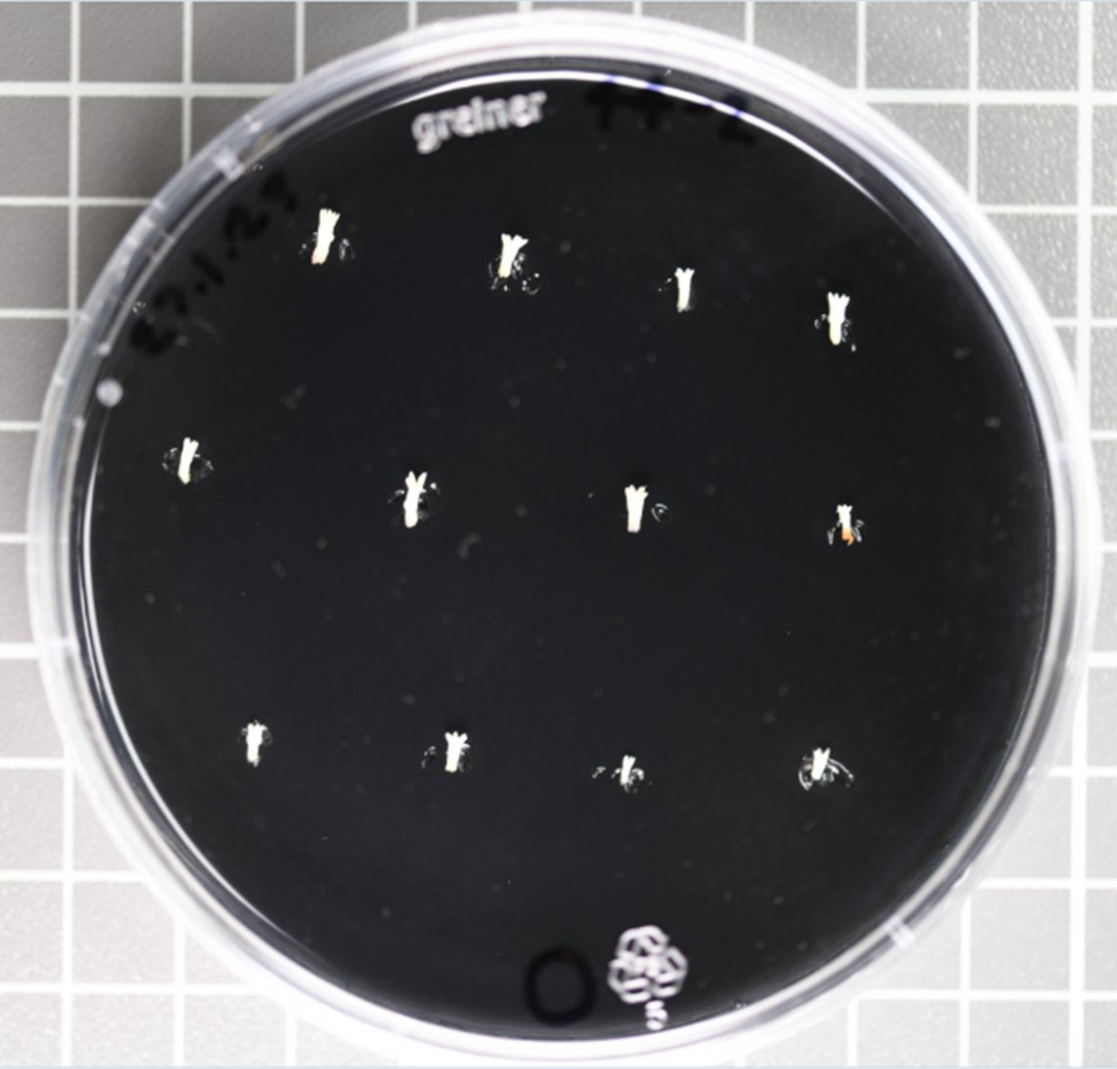
Arrangement of mature *Pinus radiata* somatic embryos on Petri plate with germination medium on the day of collection.

A total of 658 mature somatic embryos from six embryogenic cell lines of *P. radiata* were included in this study. 275 were annotated and used in training and testing from cell lines A, B, and C, with the remainder 383 from cell lines D, E, and F used as an independent set. Mature somatic embryos were produced on semi-solid culture media as previously described (Poovaiah et al., 2021; Reeves et al., 2023). All somatic embryos were collected manually with forceps and placed on germination medium (Reeves et al., 2023) in a regular pattern as illustrated in **Figure 1**. A range of morphologically normal (good) and abnormal (bad) embryos were included in this study.

### 2.2 Image acquisition

On the day of collection, lateral view images of individual mature somatic embryos were captured with a LEICA MZ FLIII stereomicroscope under 1x objective lens, and 0.8x ocular tube magnification, with an Axiocam 105 colour camera. Raw image size was 2560 x 1920 pixels, with a resulting pixel size of 4.8µm.

### 2.3 Image annotation

**Figure 2:**
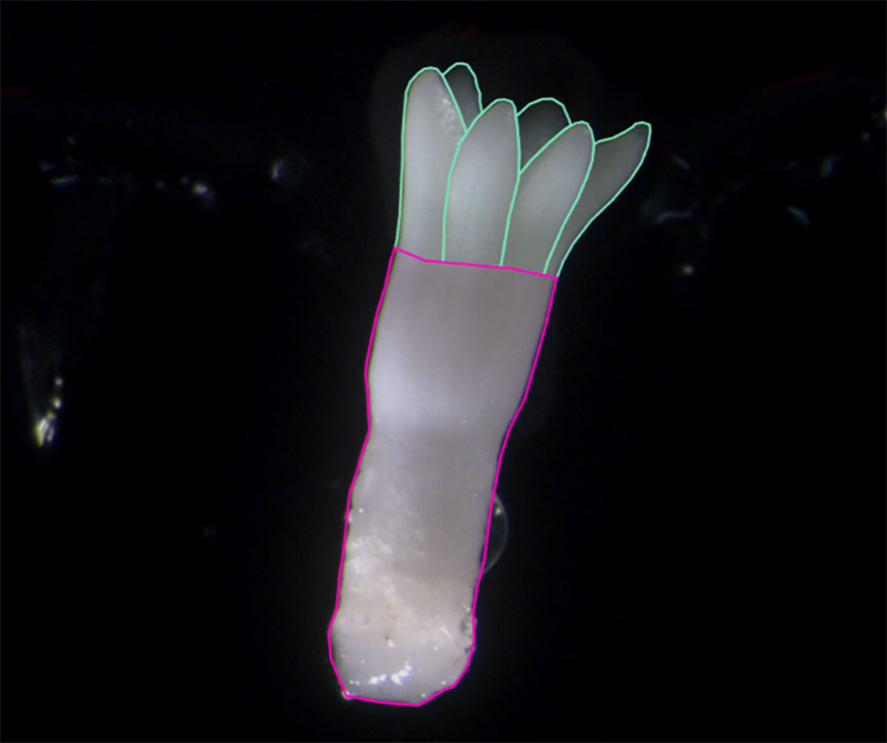
Annotated image of a *Pinus radiata* somatic embryo. The individual cotyledon instances make up the cotyledon region for semantic segmentation (outlined in green). The entire lower region is classified here as hypocotyl (outlined in pink).

The training of CNNs based models requires a variety of manually annotated images to provide the network with examples of what cotyledon and hypocotyl regions look like. Computer Vision Annotation Tool (CVAT) was used for manual image annotation of hypocotyl and individual visible cotyledons (CVAT.ai, 2022). **Figure 2** depicts an annotated embryo. For instance segmentation the annotations were exported as a CVAT COCO JSON 1.0 format. In the case of semantic segmentation, cotyledon instances were merged into a single class called cotyledon, and annotations were exported as Segmentation Mask 1.1 format which provides an individual PNG file per image with pixels coloured by their class.

The dataset of 275 colour images was randomly split into a ratio of 70:15:15 for training, validation and testing sets, respectively, resulting in 42 images in the test set (**Table 1**). 1866 polygons were annotated in total which included 1591 individual visible cotyledons (**Table 1**).

**Table 1:** Number of instances and Pinus radiata somatic embryo images for cotyledon and hypocotyl classes for training, validation, and testing sets.

	Cotyledons	Hypocotyl	Images
**Train**	1094	191	191
**Validation**	254	42	42
**Test**	242	42	42

For an additional 383 embryo images, across three different cell lines (D, E, F), we recorded the number of visible cotyledons to evaluate the model’s ability to detect cotyledons from cell lines the model had never seen before.

### 2.4 Deep learning

**Figure 3:**
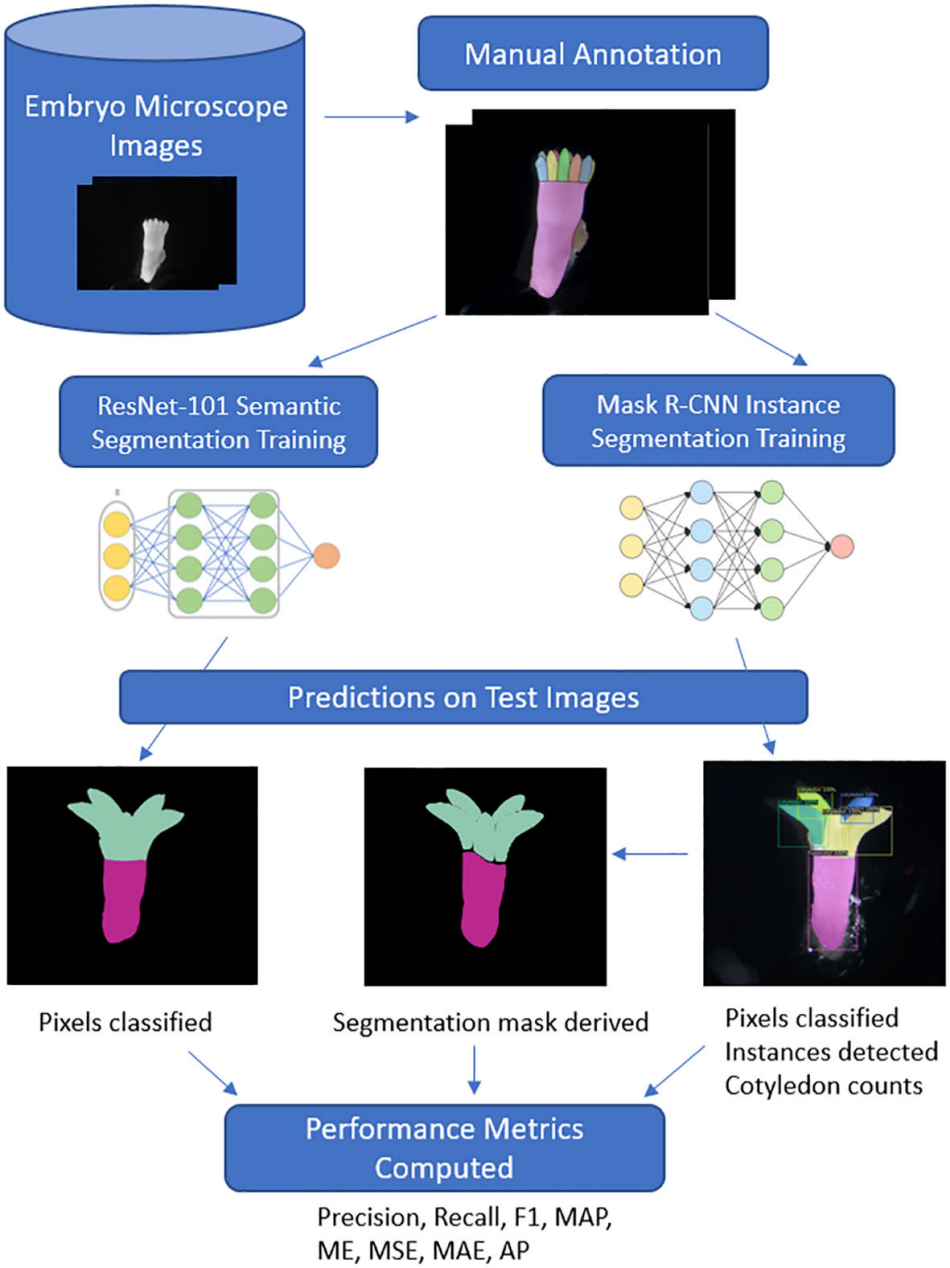
Deep learning workflow for *Pinus radiata* somatic embryo segmentation. Images are captured under a high-resolution microscope before being manually annotated to train and evaluate the two neural networks. For Mask R-CNN instance segmentation, cotyledon instance predictions are combined to derive a segmentation mask for direct comparison of pixel-wise metrics with ResNet semantic segmentation. Additionally, individual instances detected in boxes allow for cotyledon counts to be derived and a range of performance metrics are evaluated.

Deep learning instance segmentation was used to automatically segment the individual instances of hypocotyl and cotyledons allowing for automated cotyledon detection and counting. The Mask R-CNN model (He et al., 2017) was trained using open-source python library Detectron2 (Wu et al., 2019). ResNet-101 (He et al., 2016) was used as the feature extractor and Feature Pyramid Network (FPN) (Lin et al., 2017) as the decoder. We utilized a pre-trained network trained on the ImageNet dataset to help account for the relatively small dataset size. Transfer learning from ImageNet was employed to leverage the feature representations learned from this large dataset, enhancing the network’s ability to capture meaningful features on our specific task with a relatively small dataset. Fine-tuning on our target task of hypocotyl and cotyledon detection allows the model to adapt its learned features for this objective. ResizeShortestEdge, a widely used Mask R-CNN transformation, was used to train the model on different input size images with the short edge length set value to 1100 and max size value set to 1500 for model training. The network was trained on an NVIDIA RTX 3090 GPU with 24GB of memory with a batch size of 2 for 100,000 iterations (equivalent to 260 epochs) with the default learning rate of 0.001. The model which gave the highest segmentation mask mean average precision (MAP) on the validation set was used for inference on the testing set. The stochastic gradient descent optimizer was used for the network optimization. To enable a direct comparison with semantic segmentation predictions, the individual instance predictions were merged according to their class and converted to multiclass segmentation masks (**Figure 3**). In cases where there was overlap between cotyledon and hypocotyl instances, we set the hypocotyl pixels to foreground to override the cotyledon pixels as this represents a delineation between the two regions more similar to that of the data annotation method.

**Mask R-CNN detection metrics**


$$\text{MAP}=\frac{1}{C}\sum_{i=1}^{C}{\text{AP}}_{i}$$

where ***C*** is equal to the number of classes ***AP*** is the average precision per class. It is obtained by computing the area under the precision-recall curve to get ***AP*** for both classes. The mean of the *AP_i_* values across all classes gives the final mean average precision (MAP) score. This metric was only used to select the best performing instance segmentation model.

Deep learning semantic segmentation was used to automatically identify pixels belonging to the hypocotyl and cotyledon regions. The model was trained using open-source Segmentation Models PyTorch python library (Yakubovskiy, 2021). To match the encoder-decoder architecture of the Mask R-CNN network, we used ResNet-101 as the segmentation encoder and FPN as the decoder, also pretrained on the ImageNet database. Dice loss was used as the loss function to train the network and Adam was used as the network optimization function with a learning rate of 0.0001. The network was trained for 100 epochs with a batch size of 2 and the final model used for inference on the test set was the one which gave the highest IoU score on the validation set. For both models, the data augmentation techniques of horizontal flip, random crop, and random rotate were used. Data augmentation involves creating different transformations of the image-mask pairs and providing them as additional examples for the model to learn from. This forces the model to learn additional patterns and information that was not present in the raw images, improving the model’s ability to generalise to embryo or genotypes it has never seen before. Images were converted to greyscale for training both models. For a comparison with the ability of instance segmentation to do the same, the predicted cotyledon instances were merged to form the single cotyledon region mask for evaluating segmentations (**Figure 3**). **Figure 3** shows the end to end workflow of the two approaches from data annotation to testing and comparing the approaches on the test images.

Commonly used image segmentation metrics, intersection over union (IoU), precision, recall, and F1 score were used to evaluate the segmentation performance of both models. To evaluate the performance of cotyledon count predictions, we adopt similar regression metrics as the previously mentioned studies on leaf counting and compute the following regression metrics: the mean squared error (MSE), along with the difference in counts (DiC) and absolute difference in counts (ADiC) which are equivalent to the more widely known mean error (ME) and mean absolute error (MAE) respectively. A final metric, agreement percentage, was also computed, which quantifies the percentage of embryos where the model correctly detected the exact number of cotyledons. These metrics were calculated as follows:

### 2.5 Semantic segmentation metrics

Precision quantifies the proportion of true positive predictions among all positive predictions:


$$\text{Precision}=\frac{\text{TP}}{\text{TP + FP}}$$

Recall quantifies the proportion of actual positive instances that are correctly identified:


$$\text{Recall}=\frac{\text{TP}}{\text{TP + FN}}$$

where TP represent the True Positives which are the correctly predicted positive observations, FP represents the False Positives which are the incorrectly predicted positive observations and FN represents the False Negatives which are the incorrectly predicted negative observations. These are computed for every pixel.

F1-score is considered the harmonic mean of both precision and recall:


$$\text{F}1-\text{score}=2\times \frac{\text{Precision}\times \text{Recall}}{\text{Precision + Recall}}$$

**Figure 4:**
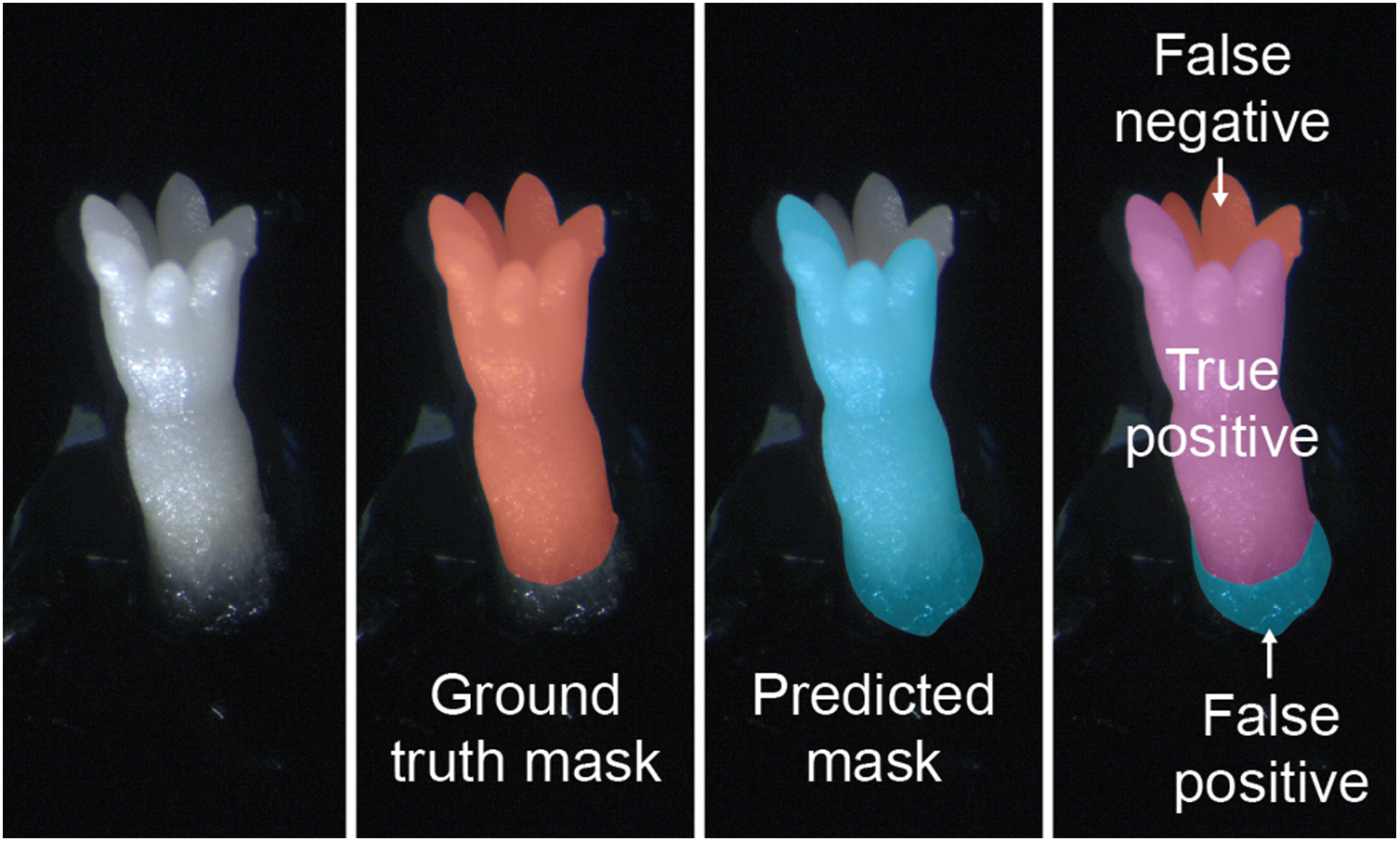
Examples of ground truth segmentation mask, predicted mask, and both together applied to images of mature somatic embryos of *Pinus radiata*. False Positive (FP), True Positive (TP) and False Negative (FN) were used to compute IoU.


IoU represents the ratio of intersected area (TP) to the combined area (TP+FP+FN) of the predicted and ground truth masks (**Figure 4**):

$$\text{IoU}=\frac{\text{Intersection}}{\text{Union}}=\frac{\text{TP}}{\text{TP + FP + FN}}$$

### 2.6 Cotyledon counting metrics

Mean Error (***ME***) calculates the average of the differences between predicted and ground truth values. 

Mean Absolute Error (***MAE***) calculates the average of the absolute differences between predicted and ground truth values.


$$MAE\;/\;ADiC\;=\sum_{i=1}^{n}\left|y_{i}-\bar{y}\right|$$

where *n* is the total number of embryo, *y_i_* is the ground truth value for embryo *i* and $\bar{y}\;$ is the predicted value.

Mean Squared Error (***MSE***) calculates the average of the squared differences between the predicted values and ground true values.


$$MSE=\frac{1}{n}\sum_{i=1}^{n}{\left(y_{i}-\bar{y}\right)}^{2}$$

Agreement percentage computes the ratio of the number of embryo where the predicted cotyledon count is equal to the actual value out of all embryo.


$$Agreement\;\%=\left(\frac{Number\;of\;embryo\;where\;predicted\;count\;is\;equal\;to\;the\;true\;count\;}{Total\;number\;of\;embryo}\right)\;\times 100$$

## 3 Results

### 3.1 Semantic segmentation

The per-class segmentation results from semantic segmentation are reported in **Table 2**. For the cotyledon class, the segmentation model achieved precision of 0.942, recall of 0.915, and F1-score of 0.929. The hypocotyl class segmentation demonstrated a precision of 0.922, recall of 0.942, and F1-score of 0.932. We also evaluated the overall segmentation accuracy using the Intersection over Union (IoU) metrics. The IoU scores for the cotyledon, and hypocotyl classes were 0.867, and 0.872 respectively. It is common for the IoU score to be lower than the other metrics due to the formula penalizing the prediction for both false positives and false negatives.

**Table 2:** Semantic segmentation evaluation metrics for each class on the test dataset of *Pinus radiata* somatic embryos.

Classes	Precision	Recall	IoU	F1
Cotyledon	0.942	0.915	0.867	0.929
Hypocotyl	0.922	0.942	0.872	0.932

The per-class semantic segmentation results from the instance segmentation approach are reported in **Table 3**. For the cotyledon class, the segmentation model achieved precision of 0.963, recall of 0.896, and F1-score of 0.928. The hypocotyl class segmentation demonstrated a precision of 0.915, recall of 0.959, and F1-score of 0.937. The IoU scores were similar to semantic results, with values of 0.866, and 0.881 for the cotyledon and hypocotyl classes respectively.

**Table 3:** Instance segmentation evaluation metrics for each class on the test dataset of *Pinus radiata* somatic embryos.

Classes	Precision	Recall	IoU	F1
Cotyledon	0.963	0.896	0.866	0.928
Hypocotyl	0.915	0.959	0.881	0.937

**Figure 5:**
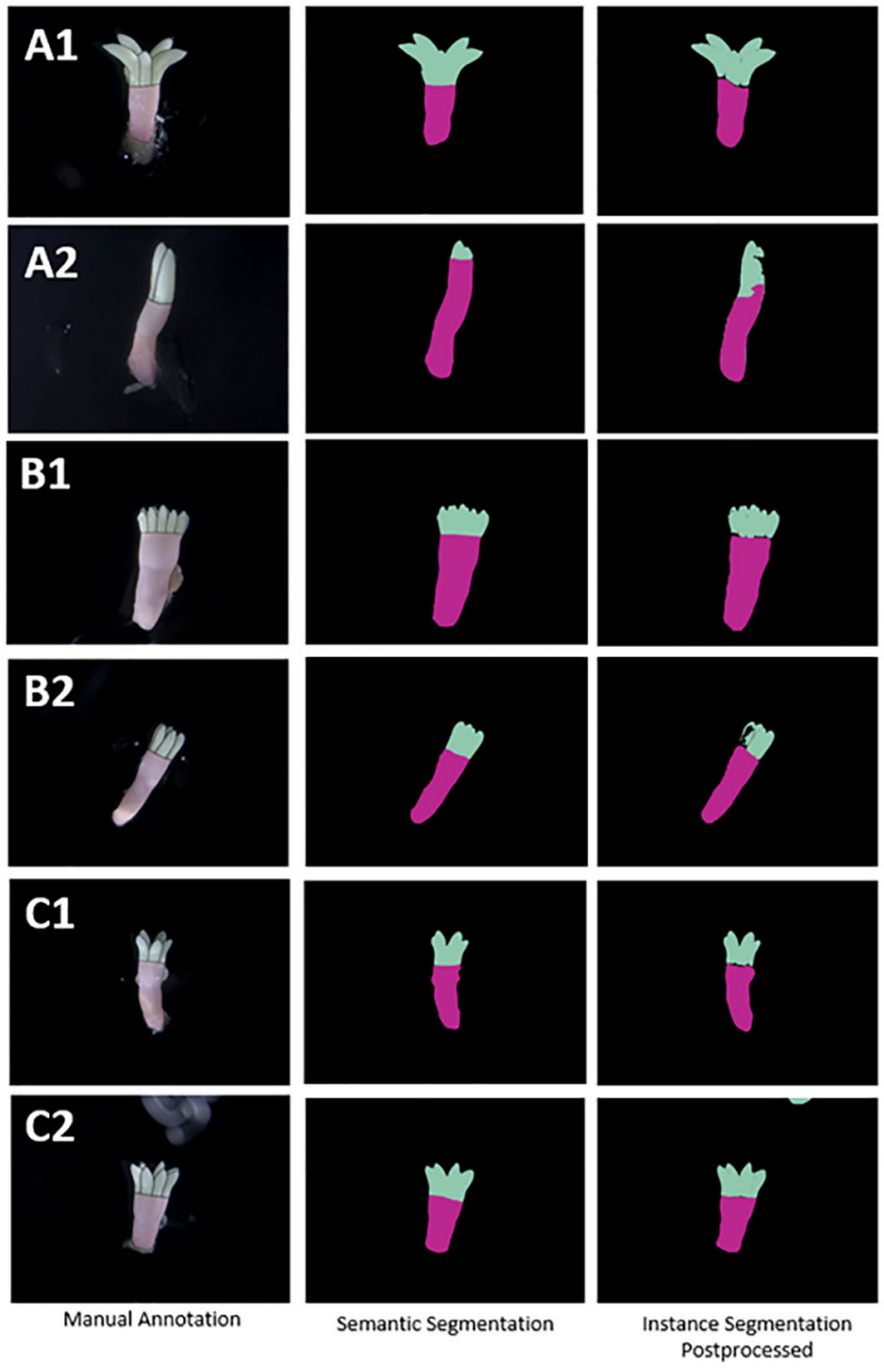
Examples of segmentation mask predictions compared with manual annotation of *Pinus radiata* somatic embryos (on original colour images) for both semantic segmentation and postprocessed instance segmentation masks for two embryos from each of the three cell lines **(A–C)** respectively.

**Figure 5** illustrates the manual annotation and both the semantic and instance segmentation prediction masks for six embryos, with two from each of the three cell lines. The semantic segmentation approach resulted in better masks (region boundaries) from a visual perspective, while the instance segmentation approach resulted in gaps between and within regions. Both approaches generally did well at segmenting the lower end of the hypocotyl (**Figure 5** B1, B2, C1 and C2). Results for both methods showed they were able to ignore residual proliferating embryogenic tissue attached to the embryo, with segmentations of embryo in A1, A2, B1 and C2 demonstrating this. Segmentation performance in the cotyledon-hypocotyl boundary was notably less accurate for instance segmentation when compared with the manual annotation, e.g. in A1 where there is a significant curve in the upper hypocotyl predicted mask. Similarly, instance segmentation commonly displayed gaps between these two regions (A1, B1, B2, C1). Apart from this, both methods showed good ability to ignore bright non-embryo noise containing regions in the image, with the left side of A2 being a clear example of this. Row C2 shows instance segmentation incorrectly detecting an artifact (a label on the petri plate lid) as a cotyledon. In one image, the instance segmentation model confused the whole embryo as being a cotyledon which results in a significantly worse segmentation mask compared to the semantic segmentation prediction. However, for this long narrow embryo, the hypocotyl region was segmented more accurately compared to the semantic segmentation mask. Row B2 highlights another image where semantic segmentation resulted in a significantly better segmentation, this time for the cotyledon region with instance segmentation resulting in gaps in the cotyledon region. Attached residual proliferating embryogenic tissue appeared on a small number of embryos, as shown at the bottom part of the embryos A1 and A2 in **Figure 5**, and the model was still able to correctly ignore these as not belonging to the embryo.

### 3.2 Instance segmentation for detection of individual instances

**Figure 6:**
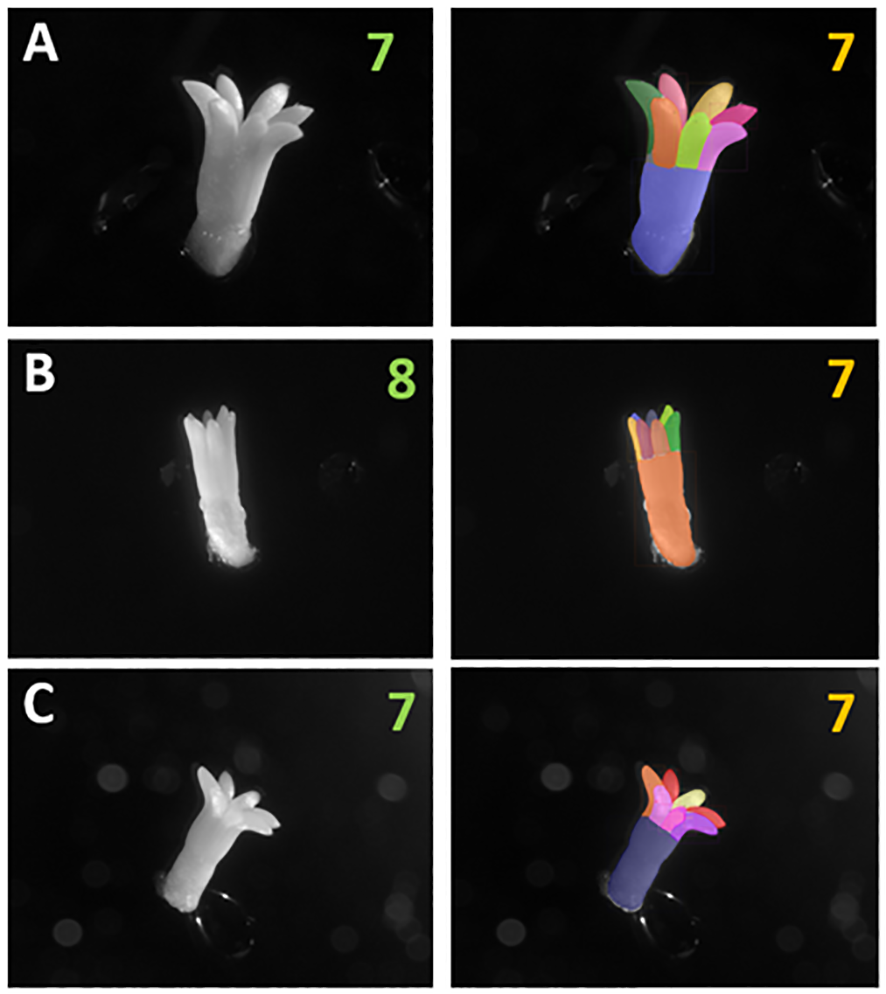
Raw images of *Pinus radiata* somatic embryos and corresponding instance segmentation masks derived from Mask R-CNN. Displaying the manually identified number of cotyledons as green text and the model detected number as orange text, for embryos from three different cell lines **(A-C)**. Different region colours represent different instances detected. The largest instance represents the hypocotyl in each image.

**Figure 6** shows a visual evaluation of instance segmentation predicted instances of the hypocotyl and cotyledons per embryo for three examples from the test set. For embryo A and C, the exact number of cotyledons were detected, and for embryo B, an error value of one was seen, as seven cotyledons were detected when there were eight manually identified. For embryo B, the network demonstrated the ability to detect small and barely visible cotyledon instances shown by the small blue and grey instances on the far side of the cotyledon region. The cotyledon that was not detected by the network is barely visible to the human eye unless zooming in. It is a small tip located on the far-right hand side of embryo B and is almost fully occluded by another cotyledon.

### 3.3 Cotyledon counts

**Figure 7:**
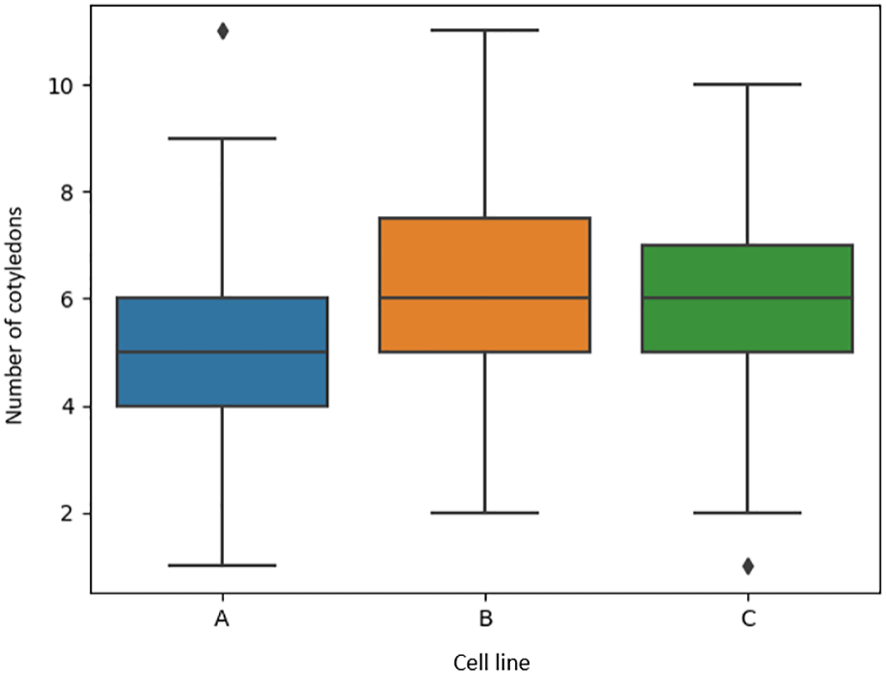
Boxplots of the number of manually identified cotyledons of *Pinus radiata* somatic embryos for cell lines **(A-C)** from microscope images.

**Figure 8:**
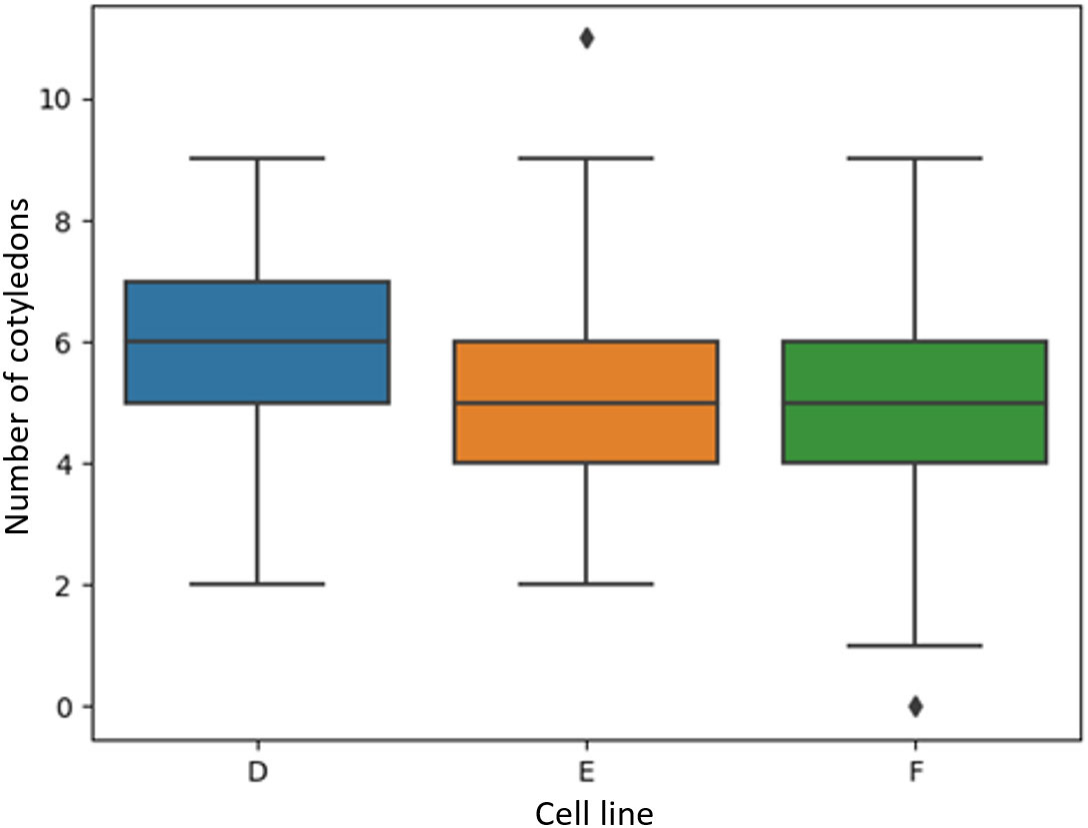
Boxplots of the number of manually identified cotyledons of *Pinus radiata* somatic embryos for cell lines D, E and F which were not used to train the model.

**Figure 7** shows the visual distribution of cotyledon counts per cell line were not skewed towards high or low counts, with four to seven cotyledons being most common. This depended on cell line with cell lines B and C having similar distributions compared to cell line A where the average cotyledon count was a little lower. Cell lines A, B, and C had a median of five, six, and six cotyledons per embryo, respectively (**Figure 7**). Overall, the minimum number per embryo was 1 and the maximum number was eleven. This shows a wide variation in the number of cotyledons for *Pinus radiata* somatic embryos. Cell lines D, E and F had median cotyledon counts of six, five, and five respectively, with non-skewed distributions and almost all embryo having at least one cotyledon and less than ten (**Figure 8**).

**Table 4** shows the validation and test set counting results from the cotyledon instances detected by the Mask R-CNN approach. The network achieved an ME of -0.19 (0.88), MAE 0.48 (0.76), and MSE of 0.81 for the validation set. For the test set, an ME of -0.31 (0.83) MAE 0.60 (0.66), and MSE of 0.69 was obtained. Small negative values for the ME show that the network had a small bias to underpredict the number of cotyledons. The test MAE score of 0.60 is a promising performance as it shows that on average, the networks error was less than 1 cotyledon per embryo. The agreement percentage was 50%, meaning exactly half of the embryos had the cotyledon count predicted correctly.

**Table 4:** Cotyledon count metrics for the 42 *Pinus radiata* somatic embryos in the validation and test datasets from cell lines A, B and C.

Data	ME (DiC)	MAE (ADiC)	MSE	Agreement %
**Validation**	-0.19 (0.88)	0.48 (0.76)	0.81	64.3
**Test**	-0.31 (0.83)	0.60 (0.66)	0.79	50.0

For mean error (ME), mean absolute error (MAE) and mean squared error (MSE), values close to zero are best. Standard deviations are shown in brackets.

For the cotyledon counts of the three unseen cell lines, the network performed similarly well, demonstrating ME scores of 0.01 (0.70), 0.05 (0.77) and -0.18 (0.91) for cell lines D, E, and F respectively (**Table 5**). MAE scores were 0.43 (0.55), 0.47 (0.61), and 0.51 (0.78), respectively. The network returned MSE scores of 0.49, 0.60 and 0.86, respectively. Agreement scores obtained were 59.4, 58.1 and 61.5, respectively, which suggests the counting approach performed better on unseen cell lines than on the test set. This could be due to the larger sample size allowing for a more comprehensive evaluation of the approach’s performance.

**Table 5:** Cotyledon count metrics for predicting cotyledon count on *Pinus radiata* somatic embryo from three cell lines which were not used to train the model.

Cell Line	n	ME (DiC)	MAE (ADiC)	MSE	Agreement %
**D**	96	0.01 (0.70)	0.43 (0.55)	0.49	59.4
**E**	191	0.05 (0.77)	0.47 (0.61)	0.60	58.1
**F**	96	-0.18 (0.91)	0.51 (0.78)	0.86	61.5

Standard deviations are shown in brackets.

## 4 Discussion

### 4.1 Embryo segmentation

Results from the convolutional neural network approaches investigated in this study have shown strong suitability for both segmentation and detection of hypocotyl and cotyledons of somatic embryos of *Pinus radiata*. Their ability to consider shape and textural visual features, in addition to the traditionally used pixel intensity information, has yielded promising results for these segmentation-based tasks.

Semantic segmentation demonstrated strong quantitative performance in segmentation of the hypocotyl and cotyledon regions as indicated by precision, recall, and F1 scores above 0.91 for all three classes on the test set. IoU values of 0.867 for cotyledon and 0.872 for hypocotyl indicate a good level of performance, but also suggests room for improvement. This is backed up by visualisation of the segmentation predictions, as we can see that sometimes the model struggles with where to segment the boundary between these two regions. This is a challenging area even for humans as it is sometimes not obvious where the cotyledons end and the hypocotyl region begins, so it is not surprising that there is a degree of confusion when the neural networks segment this region.

Visualisation of the semantic segmentation predictions on the test set show the model’s performance is comparable to the expert human annotator. The strong segmentation performance will allow for accurate automated measurements of morphological features which can be used in subsequent techniques, such as selection of high-quality embryos. Previous studies (Hamalainen and Jokinen, 1993; Chi et al., 1996; Find and Krogstrup, 2008; Le et al., 2021) performing image segmentation of somatic embryos using pixel intensity-based thresholding methods do not report segmentation metrics, nor do they distinguish between different regions of the embryo, so we are unable to provide a comparison to our results.

Instance segmentation gave similarly strong performance metrics for pixel segmentation of the regions of interest. However, performance was not as accurate as semantic segmentation when inspecting the visualized predictions, particularly in the cases showing separation between the hypocotyl and cotyledon regions. In semantic segmentation, the model learns what a general cotyledon region looks like from the training images, which is a likely reason why it performs better in the cotyledon metrics compared to instance segmentation. The instance segmentation approach is learning to detect individual cotyledon regions and thus is not able to consistently segment a smoothed lower cotyledon region as in the semantic segmentation approach. If relying on instance segmentation alone for the region identification, postprocessing steps such as filling holes within the cotyledon regions could improve accuracy by a small margin but they will not address the observed gap regions between the cotyledon region and hypocotyl. As a result, the semantic segmentation network is more suitable for embryo segmentation if accurate identification of hypocotyl and cotyledon boundaries without any gaps or holes is desired. Overall, the segmentation approaches show the ability for improved accuracy and consistency over previous approaches (Uozumi et al., 1993; Chi et al., 1996; Find and Krogstrup, 2008; Le et al., 2021) increasing the potential for large scale production of mature somatic embryos.

Although great care was taken by expert annotators, we acknowledge that there is potential for a small degree of annotation error. These labelling errors are due to the sometimes-objective task of deciding where the bottom of the cotyledon region finishes, and where the bottom of the hypocotyl region finishes. These errors can cause small confusions when training the model and are likely to be a contributor to pixel misclassifications, contributing to the performance metrics.

### 4.2 Cotyledon counts

Cotyledon counting test results (MAE=0.60, MSE=0.79) from the Mask R-CNN approach showed strong ability to count individual cotyledons. Agreement percentage score on the test dataset of 50 illustrates similar techniques can be as effective for somatic embryo when compared with previous studies in a related field, leaf counting, which achieved scores below 45 percent using deep learning (Aich and Stavness, 2017; Giuffrida et al., 2018). Additionally, similarly strong metrics on unseen cell lines highlights the model’s ability to generalize to embryos from cell lines that it has never seen before. The low degree of error, averaging less than one cotyledon per image, indicates that the number of cotyledons can be confidently used as an additional feature when performing subsequent analysis such as correlating germination success with cotyledon count, or when investigating cotyledon counts by genotype.

A limitation of our experimental approach is that microscopic image collection is laborious compared with standard lower resolution cameras. These lower resolution cameras can often be combined with robotics or fluidics to fully automate the acquisition as in Le et al. (2021). However, the network is likely to require fine-tuning or retraining to work well on images from a lower resolution source. Lower resolution cameras could reduce costs while increasing acquisition speed, allowing for large scale quantification of somatic embryos in an automated system such as the fluidics system or a robotics system with vision cameras.

In addition, we noticed the model sometimes failed to detect very small or partially occluded cotyledons, suggesting lower resolution lateral view images could be more challenging as it will reduce the area of those smaller or occluded cotyledons. In such a case, an apical view, as in Timmis et al. (2015) and Hirahara and Spencer (2007), would provide a clearer view of the cotyledons and their structure (Hirahara and Spencer, 2007). This would also allow for accurately estimating the true number of cotyledons instead of the visible number of cotyledons, which is a limitation of using lateral view images. If lower error is desired, future work could explore use of regression based CNNs for automating cotyledon counting which has proven highly successful in the plant counting literature on the CVPPP Plant Leaf Challenge dataset (Giuffrida et al., 2018; Itzhaky et al., 2018). These networks are trained to directly predict counts, and often don’t require manually annotated regions, instead, only requiring a count per image as input to train the network. This can save hours of manually labelling cotyledon instances in images.

Although instance segmentation allows for both segmentation and counting, the counts are not directly estimated and learnt by the network and are instead obtained by postprocessing the instances detected. Overall, our results indicate benefits to using both approaches with semantic segmentation enabling more complete prediction masks with less gaps and holes, and instance segmentation having the unique ability to infer accurate cotyledon counts. Future work could consider a fusion approach which combines segmentation and counting into a single neural network, with the goal of achieving accurate results for both tasks. Fan et al. (2022) used such an approach for binary image segmentation and counting of mature Arabidopsis plant leaves, achieving good results. Learning both the counts and the pixelwise segmentations in the same network has the potential to achieve optimal performance in both tasks, without having to train two separate CNNs.

Despite using less than 200 images to train the networks, we have demonstrated robust performance on an independent test set of 42 images, as well as on a further independent test set comprising 380 images from unseen cell lines. This strong performance on unseen data, which is considered the gold standard in data science, underscores the validity of our approach. Our use of ‘transfer learning’, a widely accepted and popular technique in the field, further bolsters the credibility of our methodology and its performance highlights that thousands of images are not required to achieve results similar to that of a human. However, a larger training dataset has potential to improve predictions as it provides the neural network with more examples to learn from. It would also allow for training the model from scratch instead of using a pre-trained network. These adjustments have the potential to improve both semantic and instance segmentation results, potentially boosting instance segmentation mask prediction performance to a similar level as semantic segmentation and therefore removing the need for implementing both approaches.

## 5 Conclusion

In this study we tested, for the first time, the performance of convolutional neural networks for the segmentation of *P. radiata* somatic embryos into cotyledon and hypocotyl regions. We also evaluated instance segmentation for the first time to distinguish individual cotyledons to allow for automated counting. The results demonstrated promising performance for both tasks, and highlight advantages over previous approaches, such as the ability to accurately delineate regions of similar spectral intensity by using shape and structural features learnt by the CNN. The ability to count cotyledons with a low degree of error removes the need for manual counting of cotyledons for any type of analysis such as genotype comparison, assessment of maturation protocols, unusual phenotype detection, and automated embryo sorting. Similarly, the ability for deep learning to automate the separation of the hypocotyl and cotyledon regions removes the need for manual image annotation to obtain these regional boundaries for further embryo analysis. Our approach of separating the embryo into two distinct regions provides added information for subsequent analysis such as quantification of morphological characteristics which are crucial variables for embryo sorting or predicting germination success.

Our work marks a crucial step in automating the classification of somatic embryos based on criteria like morphology, developmental stage, and genetic characteristics. By developing germination prediction models using these criteria, we can potentially reduce the cost of regenerated plantlets, making high-quality varieties more accessible to forestry owners. Automated sorting not only expedites the process but also minimizes human error, ensuring greater accuracy in selecting desired embryos. Beyond automation, these technologies reveal intricate patterns in large datasets, providing insights into factors influencing embryo development. This understanding can optimize culture conditions, enhancing success rates in somatic embryogenesis and biotechnologies. The integration of machine learning and automation accelerates traditional processes, fostering innovation in biotechnology.

Applying knowledge to automated systems for image acquisition and sorting (fluidic systems or robotics) is crucial. Further research should investigate the performance on images from lower resolution imaging systems which can easily be embedded into automated sorting systems for somatic embryogenesis. We believe these techniques could be successfully used for other coniferous species if species specific images are collected. Alternative and more recently developed neural networks, such as vision transformers (Thisanke et al., 2023) or networks which jointly learn segmentation and regression e.g (Fan et al., 2022) should also be considered as they may allow for greater accuracy and remove the need for using two separate approaches. Additionally, deep learning for computer vision is a rapidly progressing field and researchers should keep up to date with recent advancements, not limited to other applications.

## Data availability statement

The original contributions presented in the study are included in the article/supplementary material, further inquiries can be directed to the corresponding author.

## Author contributions

SD: Conceptualization, Formal analysis, Methodology, Software, Visualization, Writing – original draft, Writing – review & editing. JK: Funding acquisition, Writing – review & editing, Project administration, Resources, Supervision. TS: Data curation, Writing – original draft, Writing – review & editing, Conceptualization.
